# Normative values for singing voice handicap index – systematic review and meta-analysis^☆^

**DOI:** 10.1016/j.bjorl.2018.12.004

**Published:** 2019-02-20

**Authors:** Maria Sobol, Ewelina M. Sielska-Badurek, Ewa Osuch-Wójcikiewicz

**Affiliations:** a Medical University of Warsaw, Department of Biophysics and Human Physiology, Warsaw, Poland; b Medical University of Warsaw, Department of Otolaryngology, Warsaw, Poland

**Keywords:** SVHI, Singers, Normal voices, Meta-analysis, IDVC, Cantores, Vozes normais, Meta-análise

## Abstract

**Introduction:**

There are no official diagnostic protocols for singing voice assessment. In this publication, on the basis of a literature review, standards for the singing voice handicap index exclusively dedicated to voice disorders in singing have been given.

**Objective:**

The study aims to determine the normative values for the singing voice handicap index.

**Methods:**

The study is a systematic review and a meta-analysis. A systematic literature search was performed using PubMed to access relevant databases and to locate outcome studies. The “inclusion” criteria were as follows: English language, original papers and human studies retrospective and prospective papers, cross-sectional and case-control studies.

**Results:**

Eight articles were included for the final analysis. The normative value for the singing voice handicap index was 20.35 with a confidential range of 10.6–30.1 for a group of 729 healthy subjects whose voices were judged as normal, with an age range of 16–64 years.

**Conclusion:**

The mean normative value of the singing voice handicap index was 20.35 with the confidence levels between 10.6 and 30.1.

## Resumo

**Introdução:**

Não há protocolos diagnósticos oficiais para avaliação de voz no canto. Nesse estudo baseado em uma revisão de literatura, são fornecidos padrões para o índice de desvantagem vocal no canto exclusivamente dedicado a distúrbios vocais no canto.

**Objetivo:**

Determinar os valores normativos para o índice de desvantagem vocal no canto.

**Método:**

O estudo é uma revisão sistemática com metanálise. Uma busca sistemática da literatura foi feito no PubMed para acessar bancos de dados relevantes e para localizar estudos de desfecho. Os critérios de inclusão foram: estudos escritos em inglês, artigos originais e estudos em seres humanos, retrospectivos e prospectivos, estudos transversais e de caso-controle.

**Resultados:**

Oito artigos foram incluídos na análise final. O valor normativo para o índice de desvantagem vocal no canto foi de 20,35 com intervalo de confiança de 10,6 a 30,1 em um grupo de 729 indivíduos saudáveis cujas vozes foram consideradas normais, de 16 a 64 anos.

**Conclusões:**

O valor normativo médio do índice de desvantagem vocal no canto foi de 20,35 com intervalo de confiança entre 10,6 e 30,1.

## Introduction

Singers form a special group of professional voice users who are particularly sensitive to vocal disability and are at a high risk for voice impairment. Compared to non-singers they may have higher impact on their quality of life and have more self-reported voice problems. They are also more likely to seek medical care.^1^, ^2^, ^3^

Prior reports show that voice disorders adversely impact a patient's physical, emotional, economic and social life.^4^, ^5^, ^6^ Taking these reports into account, the World Health Organization has defined a handicap as a social, environmental or economic disadvantage. Thus, measuring a patient's dysfunction resulting from voice problems in singers is essential to provide their vocal health needs.

The most popular questionnaire that has been designed to measure the impact of voice problems in individuals is the voice handicap index (VHI)(Table 1).^7^ It was created to assess voice problems in the general population concerning mainly the speaking voice. The VHI is organized into three categories: functional, physical, and emotional, each having 10 items. From these 30 items five functional, three physical and two emotional were chosen to create VHI-10 questionnaire

**Table 1:** Questionnaire of singing voice handicap index.^9^

(1) It takes a lot of effort to sing.	Never	Almost never	Sometimes	Almost always	Always
(2) My voice cracks and breaks	Never	Almost never	Sometimes	Almost always	Always
(3) I am frustrated by my singing.	Never	Almost never	Sometimes	Almost always	Always
(4) People ask “What is wrong with your voice?”	Never	Almost never	Sometimes	Almost always	Always
(5) My ability to sing varies day to day	Never	Almost never	Sometimes	Almost always	Always
(6) My voice “gives out” on me while I am singing.	Never	Almost never	Sometimes	Almost always	Always
(7) My singing voice upsets me.	Never	Almost never	Sometimes	Almost always	Always
(8) My singing problems make me not want to sing/perform.	Never	Almost never	Sometimes	Almost always	Always
(9) I am embarrassed by my singing.	Never	Almost never	Sometimes	Almost always	Always
(10) I am unable to use my “high voice.”	Never	Almost never	Sometimes	Almost always	Always
(11) I get nervous before I sing because of my singing problems.	Never	Almost never	Sometimes	Almost always	Always
(12) My speaking voice is not normal.	Never	Almost never	Sometimes	Almost always	Always
(13) My throat is dry when I sing.	Never	Almost never	Sometimes	Almost always	Always
(14) I’ve had to eliminate certain songs from my singing/performances.	Never	Almost never	Sometimes	Almost always	Always
(15) I have no confidence in my singing voice.	Never	Almost never	Sometimes	Almost always	Always
(16) My singing voice is never normal.	Never	Almost never	Sometimes	Almost always	Always
(17) I have trouble making my voice do what I want it to.	Never	Almost never	Sometimes	Almost always	Always
(18) I have to “push it” to produce my voice when singing.	Never	Almost never	Sometimes	Almost always	Always
(19) I have trouble controlling the breathiness in my voice.	Never	Almost never	Sometimes	Almost always	Always
(20) I have trouble controlling the raspiness in my voice.	Never	Almost never	Sometimes	Almost always	Always
(21) I have trouble singing loudly.	Never	Almost never	Sometimes	Almost always	Always
(22) I have difficulty staying on pitch when I sing.	Never	Almost never	Sometimes	Almost always	Always
(23) I feel anxious about my singing.	Never	Almost never	Sometimes	Almost always	Always
(24) My singing sounds forced.	Never	Almost never	Sometimes	Almost always	Always
(25) My speaking voice is hoarse after I sing.	Never	Almost never	Sometimes	Almost always	Always
(26) My voice quality is inconsistent.	Never	Almost never	Sometimes	Almost always	Always
(27) My singing voice makes it difficult for the audience to hear me	Never	Almost never	Sometimes	Almost always	Always
(28) My singing makes me feel handicapped.	Never	Almost never	Sometimes	Almost always	Always
(29) My singing voice tires easily.	Never	Almost never	Sometimes	Almost always	Always
(30) I feel pain, tickling, or choking when I sing.	Never	Almost never	Sometimes	Almost always	Always
(31) I am unsure of what will come out when I sing.	Never	Almost never	Sometimes	Almost always	Always
(32) I feel something is missing in my life because of my inability to sing.	Never	Almost never	Sometimes	Almost always	Always
(33) I am worried my singing problems will cause me to lose money.	Never	Almost never	Sometimes	Almost always	Always
(34) I feel left out of the music scene because of my voice.	Never	Almost never	Sometimes	Almost always	Always
(35) My singing makes me feel incompetent.	Never	Almost never	Sometimes	Almost always	Always
(36) I have to cancel performances, singing engagements, rehearsals, or practices because of my singing.	Never	Almost never	Sometimes	Almost always	Always

During clinical practice, it was noticed that singers scored significantly lower on the VHI and VHI-10 compared to non-singers.^8^ Rosen et al.^2^ and Behrman et al.^3^ found that voice handicap of singers is lower than these of non-singers. It may be due to the fact that singers may be more sensitive to voice changes. Moreover, non-singers voice problems may differ from those of singers. The VHI may not be sensitive enough for singers, as it is focused on the speaking voice. This observation led to the development of the second index: the singing voice handicap index and its shortened counterpart SVHI-10 for singers with voice problems. This index was developed and validated on performers by Cohen et al. in 2007.^9^ The SVHI questionnaire consists of 36 items: physical, emotional, social and economic individually graded on a five-point Likert scale (0 – never, 1 – almost never, 2 – sometimes, 3 – almost always, 4 – always see Appendix) to range from 0 to 144. It was proved that the SVHI can be used to measure treatment outcomes in singers.^10^ A higher score in the SVHI indicates more singing voice handicap. The SVHI compared with the VHI questionnaire is more sensitive to singers with voice disorders.^10^

The main propose of this study was to determine the normative values for the SVHI among singers whose voice were judged as normal.

## Materials and methods

Studies included in this research were selected from a systematic search of literature in PubMed. Studies published up to 15 April 2018 were included. The “inclusion” criteria were as follows: English language, original papers and human studies retrospective and prospective papers, cross-sectional and case-control studies. The ‘exclusion’ criteria were as follows: median of SVHI, mean value of SVHI without standard deviation. We did not consider case reports. The screening of the results was based on the words and phrases: singing voice handicap index (SVHI), singers, professional voice users, normal voices. Unpublished reports and those without peer-review evaluations, abstracts, or incomplete text were not considered. Authors were not contacted. Two reviewers, the first and second author, assessed each abstract for potential inclusion and reached a consensus for the final article to be included in the review.

### Eligibility criteria

The systematic review was conducted using the PRISMA guidelines.^11^ Specific requirements are listed as follows:

*Patient/population*: Professional voice users, adults with normal voices and no identifiable vocal fold pathology, all age groups.

*Limits used*: Human subject studies, studies published in English.

**Figure 1:**
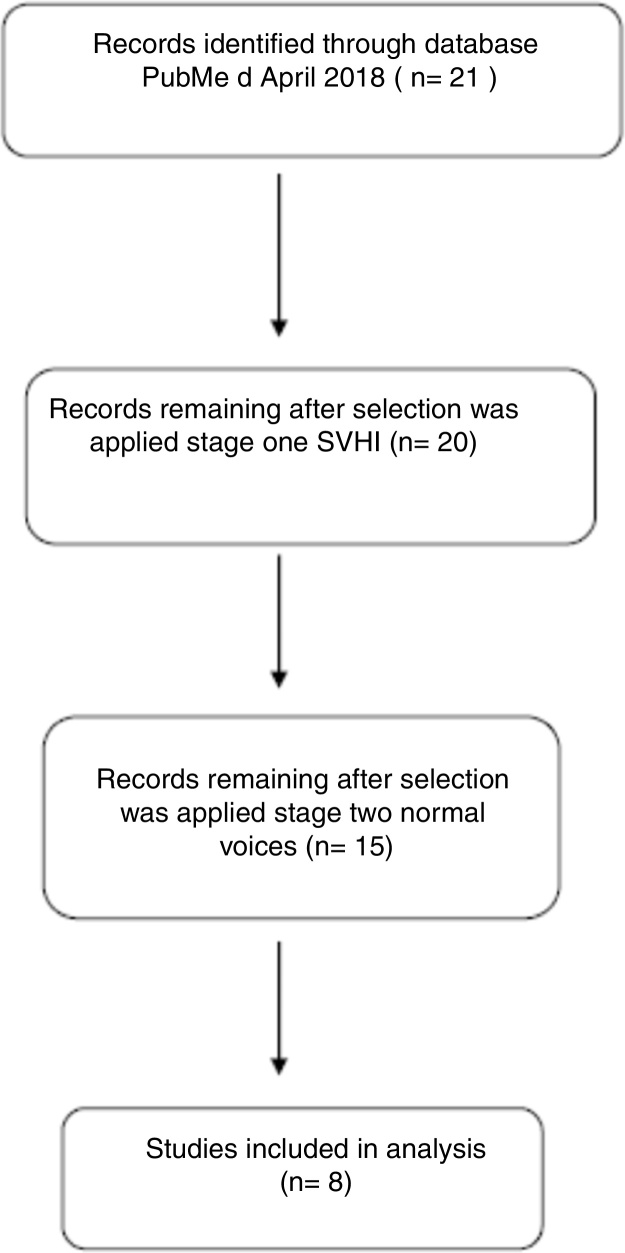
Flow chart for inclusion of articles.

*Search string*: Search string details are shown in Fig. 1.

### Statistical analysis

Statistical analysis was performed using the Statistica 13 package. The statistical heterogeneity was assessed using the inconsistency index *I*^2^ measure. The analysis was conducted with a fixed-effects model and the standardized mean difference with 95% Confidence Intervals (95% CI).

## Results

Our search strategy identified 21 articles among the PubMed database. After screening using the phrases: singing voice handicap index (SVHI), adults and normal voices, and professional voice users, eight studies were identified^12^, ^13^, ^14^, ^15^, ^16^, ^17^, ^18^, ^19^ and therefore included. In Table 2 e characteristics of the studies are summarized. In the meta-analysis we only considered professional singers who identified themselves as being healthy, which was defined as having no known vocal problems.

**Table 2:** The SVHI scores and standard deviations.

Study	SVHI control group mean (SD)	Numbers of subject
Lorenz et al.^12^	15.0 (12.7)	130
Garcia-Lopez et al.^13^	28.43 (18.58)	81
Baracca et al.^14^	29.26 (25.72)	117
Gunjawate et al.^15^	19.81 (10.74)	84
Lee et al.^16^	19.84 (12.84)	90
Castelblanco et al.^17^	22.4 (16.33)	47
Sielska-Badurek et al.^18^	19.4 (11.2)	57
Denizoğlu et al.^19^	21.8 (18.5)	123

Most of the studies were from Europe (*n* = 6), followed by Egypt (*n* = 1), and India (*n* = 1).

**Figure 2:**
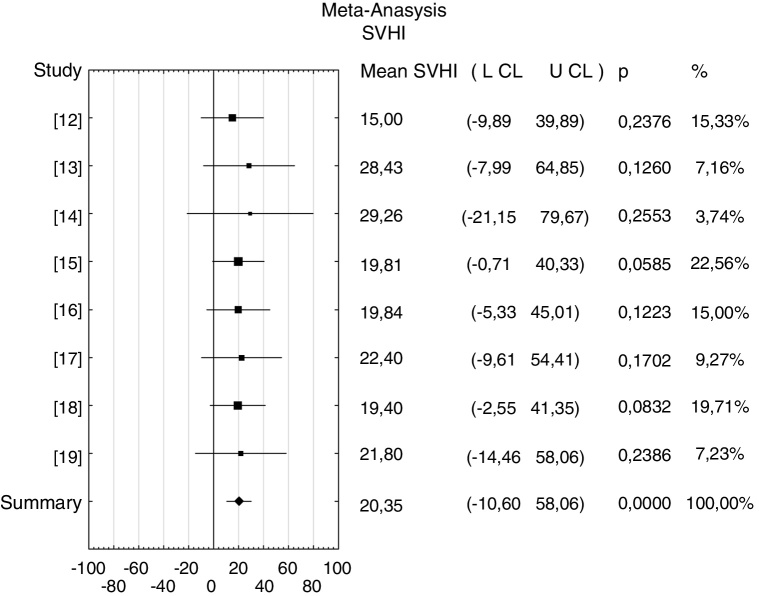
Forest plot of the SVHI score, *p*-value indicating level of statistical significance.

Data analysis was conducted using meta-analysis. The range of age was 16–64 years. The received mean normative value of the SVHI for a group of 729 healthy professional voice users was 20.65 with 95% confidence levels of 10.6–30.1 (Fig. 2). A heterogeneity for the different studies included in the meta-analysis was *I*^2^ = 0% so the fixed effect model was used.

## Discussion

The purpose of this study was to find the normative value for the SVHI for a large population of healthy professional singers. Singers represent a unique group of professional voice users. They are at risk of developing voice problems that affect not only their speaking but also their singing voice. To maximize treatment of this special group of people, it is essential to understand how voice problems impact them. Although the VHI was applied to evaluate singing voice,^8^ it was noticed that it had poor sensitivity to evaluate singing voice problems. As a result, there was a need to create a more specific self-reported instrument – the singing voice handicap index. It is a specific questionnaire to asses singing voice under emotional, social, physical and economic domains. It was shown^10^ that SVHI is sensitive to clinical changes and helps to identify specific singing problems.

Validation and adaptation of SVHI was done in many languages: English,^9^ German,^12^ Italian,^14^ Turkish,^19^ Spanish^13^ and Polish.^18^ Most of the studies considered small groups of healthy participants so it is very difficult to give a credible score of normative voices for singers. To receive a more appropriate score we considered a relatively large group of healthy individuals without voice or hearing complaints from multicentre studies. To do this we did a systematic literature search of the PubMed electronic database. Eligibility criteria included type of publication, participant characteristic (for our analysis we chose only healthy subjects) and report of outcomes.

The received mean normative value of the SVHI for a group of 729 healthy singers was 20.65 with 95% confidence levels from 10.6 to 30.10. The normal value of VHI is known to be between 0 and 30, however, itshould be noted that a result above 30 will not always be synonymous with the existence of actual voice disorders.^20^ Our result is in agreement with Renk et al.^21^ who showed that the mean value of VHI-10 (12.1) scores of singers were significantly lower than those of SVHI-10 (20.4). They noticed that these two groups ranked differently in 10 statements. In addition, when Murry et al. changed statements from “my voice” to “my singing voice” the group of singers judged their voice complaints as more severe and it probably caused some differences in the VHI-10 and SVHI-10 scores. The reason behind higher results of SVHI score can be thought as a general complaint of singers about their voice. The professional voice users as singers have also specific complaints that rate only their singing voice.

In performed meta-analysis we considered only articles that we found in PubMed. We considered only English language papers in which results of SVHI scores were presented as mean ± standard deviation. We excluded all data given as a median of SVHI with range or mean value of SVHI without standard deviation, which limited the numbers of the results that we could use.

There are no official diagnostic protocols for singing voice assessment. In this publication, on the basis of a literature review, standards for a tool exclusively dedicated to voice disorders in singing have been given. Setting standards for the SVHI – means that the questionnaire will become a much more sensitive and specific tool for detecting voice disorders in singing. In addition, it will be able to be used by laryngologists, phoniatrists, speech therapists, and singing pedagogues.

## Conclusion

In the performed meta-analysis the mean normative value of the singing voice handicap index for a group of 729 healthy singers (aged 16–64) was 20.35. The singing voice handicap index confidence level was between 10.6 and 30.1. In the future, it would be wise to carry out a subordinate analysis allowing determining the singing voice handicap index range for mild voice disorders or severe voice disorders in singing.

## Conflicts of interest

The authors declare no conflicts of interest.

## References

[1] Phyland D.J., Oates J., Greenwood K.M. (1999). Self-reported voice problems among three groups of professional singers. J Voice.

[2] Rosen C.A., Murry T. (2000). Voice Handicap Index in singers. J Voice.

[3] Behram A., Stulica L., He T. (2004). Factors predicting patient perception of dysphonia caused by benign vocal fold lesions. Laryngoscope.

[4] Cohen S.M., Dupont W.D., Courey M.S. (2006). Quality-of-life impact of non-neoplastic voice disorders; a meta-analysis. Ann Otol Rhinol Laryngol.

[5] Benninger M.S., Ahuja A.S., Gardner G., Grywalski C. (1998). Assessing outcomes for dysphonic patients. J Voice.

[6] Roy N., Merrill R.M., Gray S.D., Smith E.M. (2005). Voice disorders in general population; prevalence, risk factors, and occupational impact. Laryngoscope.

[7] Jacobson B.H., Johnson A., Grywalski A., Silbergleit A., Jacobson G., Benninger M.S. (1997). The voice handicap index: development and validation. Am J Speech Language Pathol.

[8] Murry T., Zschommler A., Prokp J. (2009). Voice handicap in singers. J Voice.

[9] Cohen S.M., Jacobson B.H., Garrett C.G., Noordzij J.P., Stewart M.G., Attia A. (2007). Creation and validation of the Singing Voice Handicap Index. Ann Otol Rhinol Laryngol.

[10] Cohen S.M., Witsell D.L., Scearce L., Vess G., Banka C. (2008). Treatment responsiveness of the singing voice handicap index. Laryngoscope.

[11] Moher D., Liberati A., Tetzlaff J., Douglas G.A. (2009). The PRISMA Group Preferred reporting items for systematic reviews and meta-analysis: the PRISMA statement. J Clin Epidemiol.

[12] Lorenz A., Kieber B., Butner M., Fuchs M., Murbe D., Richter B. (2013). Validierung des singing voice handicap index in der deuchenFassung. HNO.

[13] Garcia-Lopez I., Nunez-Batalla F., Gavilian Bouzas J., Gorriz-Gil C. (2010). Validation of the Spanish version of the voice handicap index for vocal singing (SVHI). Acta Onorrinolaringal Esp.

[14] Baracca G., Cantarella G., Forti S., Pignataro L., Fussi F. (2014). Validation of the Italian version of the singing voice handicap index. Eur Arch Otorhinolaryngol.

[15] Gunjawate R., Aithal V.U., Guddattu V., Bellur R. (2017). Adaptation and validation of the Kannada version of the singing voice handicap index. J Voice.

[16] Lee AhR, Sim H.S. (2013). The Korean version of the singing voice handicap index. Commun Sci Dis.

[17] Castelblanco L., Habib M., Stein D.J., de Quadros A., Cohen S.M., Noordzij J.P. (2014). Singing voice handicap and videostrobolaryngoscopy in healthy professional singers. J Voice.

[18] Sielska-Badurek E.M., Sobol M., Cioch A., Osuch-Wójcikiewicz E., Rzepakowska A.M., Niemczyk K. (2017). Adaptation and validation of the Singing Voice Handicap Index into Polish. Clin Otolaryngol.

[19] Denizoğlu İ.İ., Şahin M., Kazancıoğlu A., Dağdelen Z., Akdeniz S., Oğuz H. (2016). Validation and reliability of Turkish Singing Voice Handicap Index. Kulak Burun Bogaz Ihtis Derg.

[20] Karlsen T., Grieg A.R.M., Heimdal J.-H., Aarstad H.J. (2012). Cross-cultural adaptation a translation of the Voice Handicap Index into Norwegian. Folia Phoniatr Logop.

[21] Renk E., Sulica L., Grossman Ch, Gorges J., Murry T. (2017). VHI-10 and SVHI-10 differences in singers’ self-perception of dysphonia severity. J Voice.

